# Longitudinal Change of HBsAg in HBeAg-negative Patients with Genotype B or C Infection

**DOI:** 10.1371/journal.pone.0055916

**Published:** 2013-02-20

**Authors:** Tung-Hung Su, Chun-Jen Liu, Tai-Chung Tseng, Chen-Hua Liu, Hung-Chih Yang, Chi-Ling Chen, Pei-Jer Chen, Jia-Horng Kao, Ding-Shinn Chen

**Affiliations:** 1 Department of Internal Medicine, National Taiwan University Hospital, Taipei, Taiwan; 2 Graduate Institute of Clinical Medicine, National Taiwan University College of Medicine, Taipei, Taiwan; 3 Hepatitis Research Center, National Taiwan University Hospital, Taipei, Taiwan; 4 Division of Gastroenterology, Department of Internal Medicine, Buddhist Tzu Chi General Hospital Taipei Branch, Taipei, Taiwan; 5 Department of Microbiology, National Taiwan University College of Medicine, Taipei, Taiwan; The University of Hong Kong, Hong Kong

## Abstract

**Background & Aims:**

Quantitative HBsAg has been recognized to assist in the management of chronic hepatitis B virus (HBV) infection. However, its role in disease monitoring of HBeAg-negative patients remains unclear. We aimed to investigate the longitudinal HBsAg change in HBeAg-negative carriers with HBV genotype B or C infection.

**Methods:**

This is a retrospective cohort study conducted in a university hospital. Treatment-naïve HBeAg-negative carriers followed for more than 3 years were recruited. Their hepatitis activities were categorized by longitudinal HBV-DNA levels into high viral-load (HVL: HBV-DNA >/ = 2000 IU/mL persistently), low viral-load (LVL: HBV-DNA <2000 IU/mL persistently) and fluctuated viral-load (FVL: HBV-DNA between HVL and LVL). The baseline and end-of-follow-up (EOF) HBsAg levels were quantified for analyses.

**Results:**

We recruited 187 patients with a median follow-up of 8 years. LVL patients had a significantly lower HBsAg at baseline and EOF and a significantly greater annualized HBsAg decline compared with the FVL and HVL. The longitudinal HBsAg change was independent of genotype B or C. The lower baseline HBsAg level predicted the HBsAg decline and HBsAg loss, whereas the higher baseline HBV-DNA predicted the hepatitis flare. A baseline HBsAg <50 IU/mL predicted subsequent HBsAg loss with a sensitivity of 82% and specificity of 67%. The annualized HBsAg decline appeared non-linear, and accelerated as the HBsAg level lowered (0.054, 0.091, 0.126 log_10_ IU/mL in patients with baseline HBsAg >1000, 100–999, <100 IU/mL, respectively, P for trend = .014).

**Conclusions:**

In genotype B or C HBeAg-negative carriers, baseline HBsAg levels correlate with future disease activities and help to predict HBsAg decline or loss. Inactive carriers with lower baseline HBsAg levels have a greater and accelerating HBsAg decline over time, regardless of HBV genotypes.

## Introduction

The natural history of hepatitis B virus (HBV) carriers who acquire the virus in early life can be divided into 4 dynamic phases based on virus-host interactions, including: immune tolerance, immune clearance, inactive carrier state and reactivation phase. [1], [2] After hepatitis B e antigen (HBeAg) seroconversion, patients usually enter the inactive state, with low viral load and normal alanine aminotransferase (ALT) level. However, a certain proportion of inactive carriers may experience HBV reactivation, which accelerates the disease progression to end-stage liver disease, including cirrhosis and hepatocellular carcinoma (HCC). [3] Therefore, patients in the HBeAg-negative phase should still receive regular monitoring of the hepatitis activity.

The introduction of HBsAg quantification has provided a new diagnostic tool in the management of chronic hepatitis B (CHB), such as the prediction of disease activity, HBsAg loss, and development of HCC in the natural history,[4]–[9] as well as the responses to treatment.[10]–[12] Several cross-sectional studies have shown the dynamic change of HBsAg levels during the natural course of HBV infection: higher in the immune tolerance and clearance phase, lower in the inactive phase and increases again in the reactivation phase.[13]–[16] In an European study with 3 years of longitudinal follow-up of 209 genotype D HBeAg-negative patients, HBsAg levels correlated with disease activity and helped to predict the inactive carrier state. [8] In another longitudinal study on 68 genotype B or C HBeAg-negative patients, HBsAg levels tended to be higher in the active carriers. [14] These lines of evidence suggest that, HBsAg quantification can be utilized for monitoring in patients with chronic hepatitis B infection because of its dynamic change and complementary role to HBV-DNA.

However, most of the previous studies looking at the clinical significance of HBsAg level were of cross-sectional design with a limited follow-up duration or case numbers. The definition of disease activities mostly relied on the short-term observation of HBV-DNA; however, the HBV-DNA tended to fluctuate overtime. Therefore, larger studies with longer observation period are still needed for validation, especially in Asian countries where most chronic HBV infections are acquired early in life. To that end, we conducted a study to investigate the longitudinal HBsAg change in HBeAg-negative patients with genotype B or C infection, and to explore the role of baseline HBsAg level in predicting disease progression in this special clinical setting.

## Materials and Methods

### Patient Cohort

**Figure 1:**
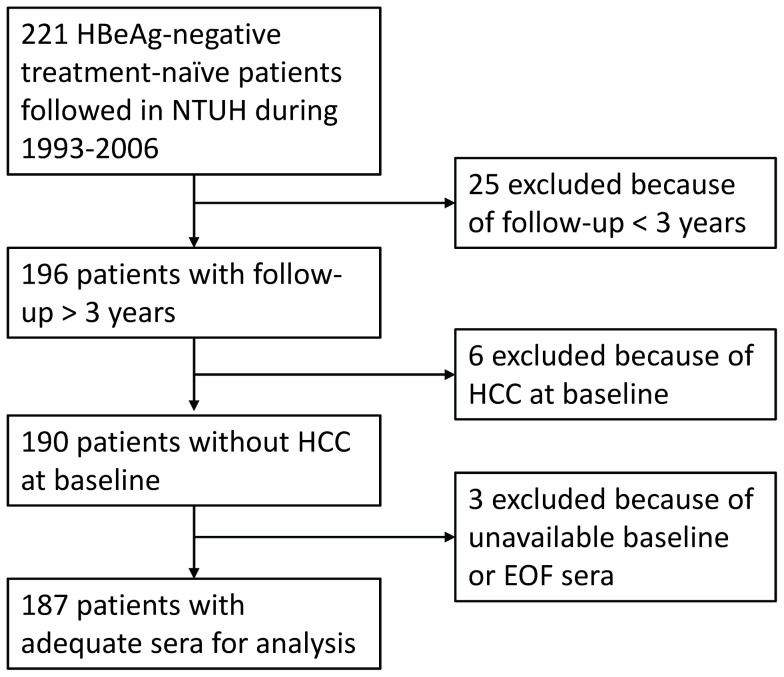
The flow chart of patient enrollment. EOF = end-of-follow-up, HCC = hepatocellular carcinoma.

A total of 187 HBeAg-negative carriers were recruited from the Gastroenterology Clinics in National Taiwan University Hospital between 1993 and 2006. All of them fulfilled the criteria of >20 years old, HBsAg-positive and HBeAg-negative >6 months, and had never received anti-HBV treatment. Patients should have available baseline and end-of-follow-up (EOF) sera stored at −20°C refrigerators in the Hepatitis Research Center and a follow-up duration >3 years. Patients with malignancy, autoimmune disease, hemochromatosis, Wilson’s disease, alcohol intake >40 gm/day, and HIV, HCV or HDV co-infection were excluded. At first, 221 patients fulfilled the inclusion criteria, but 34 were excluded due to follow-up duration <3 years in 25 patients, HCC at baseline in 6 patients and unavailable baseline or EOF serum sample in 3 patients [Figure 1]. The study conformed to the ethical guidelines of the 1975 Declaration of Helsinki and was approved by the Institutional Review Board of the National Taiwan University Hospital. Written informed consents were obtained from all patients at enrollment.

### Data Collection

Throughout the follow-up period, liver function tests and alpha-fetoprotein were assayed every 6 months if ALT levels were within normal limits (</ = 40 U/L) and at least every 3 months if ALT levels were elevated. Pertinent clinical information was collected from the patient’s medical records. The EOF was defined as the date of the last visit, the start of anti-HBV therapy or December 31, 2010, whichever came first.

### HBV Serological and Virological Assays

Serum HBeAg, anti-HBe, anti-HCV and anti-HDV were tested by using commercial kits. (Abbott Laboratories, Abbott Park, IL, USA). Serum HBsAg titer was quantified by Architect HBsAg QT assay (Abbott Laboratories) with a diagnostic range from 0.05 IU/mL to 250 IU/mL. Serial 1∶100 to 1∶1000 dilutions were performed if the HBsAg >250 IU/mL according to the manufacturer’s instructions. The HBsAg levels were quantified in all cases at baseline, and at EOF. Among them, 165 patients with available first-year sera were also quantified for HBsAg.

Serum HBV-DNA was extracted from 200 µL of serum by QIAmp DNA Blood Mini Kit (QIAGEN Inc, Valencia, CA, USA) and quantified by a real-time PCR amplification assay using LightCycler (Roche Diagnostics, Basel, Switzerland) with a detection sensitivity of 20 IU/mL. [17] The HBV genotype was determined by the the melting curve analysis followed by the real-time PCR. [17].

### Definition of HBV Hepatitis Activity

Because HBV-DNA levels fluctuate with time, it is inadequate to define the disease activity by a short period of observation (eg. 1 year) of HBV-DNA level. In order to know the true disease activities, we classified these HBV carriers according to their longitudinal HBV-DNA level: (1) high viral-load (HVL): HBV-DNA >/ = 2000 IU/mL persistently; (2) low viral-load (LVL) HBV-DNA level <2000 IU/mL persistently; (3) fluctuated viral-load (FVL): HBV-DNA level between HVL and LVL during follow-up.

HBsAg loss was defined as two consecutive HBsAg levels of <0.05 IU/mL at least 6 months apart. Hepatitis flare was defined as ALT >5 times of the upper normal limit (</ = 40 U/L) with a concomitant serum HBV-DNA level >2000 IU/mL, [18] at least 6 months after inclusion. Cirrhosis was defined by histological or ultrasonographic findings (two consecutive examinations 6 months apart for confirmation) along with clinical features of splenomegaly, esophageal varices, or ascites. [19] HCC was diagnosed either by histology/cytology or by typical dynamic imaging study (CT, MRI or angiography) plus a serum alpha-fetoprotein level >200 ng/mL. [20].

### Statistics

Continuous variables were expressed as median (25–75th percentile) and categorical variables were expressed as number (percentage) as appropriate. The HBsAg, HBV-DNA and HBV-DNA/HBsAg levels were logarithmically transformed for analysis. For those with HBsAg or HBV-DNA level below the detection limit (0.05 IU/mL for HBsAg and 20 IU/mL for HBV-DNA), an arbitrary value of 0.025 IU/mL for HBsAg and 10 IU/mL for HBV-DNA were assigned for analysis. Differences between subgroups were analyzed using one-way ANOVA tests or Student’s t test as appropriate. Categorical variables were compared by the Chi-square test as appropriate. The decline of HBsAg and HBV-DNA were calculated by the value at baseline – value at EOF. Paired t test was used to compare the decline of HBsAg and HBV-DNA levels between the baseline and EOF. The annualized rate of HBsAg decline was computed by dividing the HBsAg decline by individual duration of follow-up. Nonparametric trend test was used for trend across ordered groups. Receiver Operating Characteristic (ROC) analysis was used for a cut-off HBsAg level to predict HBsAg loss in the LVL group. The statistical analysis was performed by STATA (version 11.0; Stata Corp, College Station, TX, USA). All tests were 2-sided and a p value <0.05 was considered significant.

## Results

### Baseline and End-of-follow-up HBsAg and HBV-DNA Levels Differed Significantly between Active and Inactive Carriers

The baseline and EOF data of our patients are shown in Table 1. The distribution of HBV genotypes was mostly B (126/166, 76%), followed by C (38/166, 23%), B+C (2/166, 1%) and was undetermined in 21 patients because of low viral-load. The median follow-up duration was 8 years. Two patients developed HCC during the longitudinal follow-up in the HVL and FVL groups, respectively.

**Table 1:** Characteristics of HBeAg-negative HBsAg carriers according to their hepatitis activities.

Characteristics	Low viral-load	Fluctuated viral-load	High viral-load	P value
	n = 43	n = 114	n = 30	
Age at enrollment	38 (33, 48)	43 (36, 51)	47 (41, 55)	.004
Male, n(%)	25 (58)	78 (68)	16 (53)	.216
Genotype B, n(%) n = 166	19 (73)	81 (74)	26 (87)	.543
Baseline				
ALT (U/L)	22 (14, 37)	30 (20, 45)	40 (25, 66)	<.001
HBsAg (log_10_ IU/mL)	2.84 (1.54, 3.34)	3.43 (2.85, 3.77)	3.37 (3.01, 3.64)	<.001
HBV-DNA (log_10_ IU/mL)	1.77 (1.00, 2.82)	3.43 (2.48, 4.08)	4.78 (3.85, 5.85)	<.001
HBV-DNA/HBsAg	−0.51 (−1.64, 0.58)	0.09 (−0.88, 1.24)	1.60 (0.77, 2.28)	<.001
Liver cirrhosis, n(%)	2 (5)	6 (5)	4 (13)	.239
End-of-follow-up				
ALT (U/L)	22 (18, 37)	34 (22, 56)	48 (30, 121)	.002
HBsAg (log_10_ IU/mL)	1.31 (−0.74, 2.90)	2.92 (1.79, 3.49)	3.13 (2.54, 3.54)	<.001
HBV-DNA (log_10_ IU/mL)	1.00 (1.00, 2.36)	3.31 (2.53, 4.65)	5.05 (4.39, 6.10)	<.001
HBV-DNA/HBsAg	0.19 (−0.43, 2.22)	1.15 (0.29, 2.28)	1.98 (1.46, 3.01)	<.001
Liver cirrhosis, n (%)	2 (5)	13 (11)	8 (27)	.017
Number of HBV DNA exams	4 (3, 5)	6 (4, 8)	7 (3, 7)	<.001
Percentage of HBV-DNA <2000 IU/mL	100 (100, 100)	50 (33, 67)	0 (0, 0)	<.001
Duration of FU, year	9.7 (4.9, 12.2)	8.7 (4.7, 11.8)	5.2 (4.3, 7.1)	.007

Data are expressed as median (25th, 75th quartile) or number (percentage) as indicated.

The baseline characteristics were comparable in terms of gender, genotype, and percentage of liver cirrhosis among three groups [Table 1]. The baseline HBsAg and HBV-DNA levels were significantly lower in the LVL group compared with the FVL and HVL groups (HBsAg: 2.84, 3.43 and 3.37 log_10_ IU/mL, P<.001; HBV-DNA: 1.77, 3.43 and 4.78 log_10_ IU/mL, P<.001). Among them, HBsAg was quantified in 165 patients (LVL/FVL/HVL: 39/101/25) with available first-year sera. The median first-year HBsAg were 2.59, 3.29 and 3.22 in the LVL, FVL and HVL groups, respectively. Compared with the FVL and LVL groups, the HVL group had significantly higher ALT, HBsAg and HBV-DNA levels at EOF. The HBV-DNA/HBsAg ratios were significantly higher in active carriers both at baseline and EOF (all P<.001).

### Long-term Events of the Individual Group

The long-term events of each group were compared in Table 2. The maximal ALT level during follow-up was significantly higher in the HVL group (median: 168 U/L). A significant HBsAg decline was observed in LVL, FVL and HVL groups when compared with the baseline level (0.99, 0.52 and 0.20 log_10_ IU/mL, respectively). The decline of HBV-DNA levels were insignificant in 3 groups (P = .248). There was no hepatitis flare in the LVL group, compared with 16% and 30% in the FVL and HVL groups, respectively (P = .001). A significant trend of increasing cirrhosis at EOF was observed in FVL and HVL groups (11% and 27%, respectively) compared with LVL group (5%, P = .017) [Table 1]. About 43% of patients in the HVL group finally received anti-viral therapy, which was significantly higher than the FVL and LVL groups (17% and 5%, respectively). Nineteen patients (10%) achieved HBsAg loss during the follow-up and 56% of them developed anti-HBs (>10 mIU/mL) spontaneously.

**Table 2:** Long-term events of groups according to their hepatitis activities.

Characteristics	Low viral-load	Fluctuated viral-load	High viral-load	P value
	n = 43	n = 114	n = 30	
Maximal ALT level (U/L)	41 (29, 75)	70 (39, 162)	168 (113, 246)	.006
HBsAg decline (log_10_ IU/mL)	0.99 (0.26, 2.05)†	0.52 (0.07, 1.06)†	0.20 (−0.04, 0.53)*	.004
Annualized HBsAg decline (log_10_ IU/mL)	0.101 (0.039, 0.203)	0.067 (0.008, 0.162)	0.040 (−0.008, 0.101)	.006‡
HBV-DNA decline (log_10_ IU/mL)	0 (0, 1.22)	−0.09 (−1.69, 1.34)	−0.54 (−1.43, 1.09)	.248
Hepatitis flare, n(%)	0 (0)	18 (16)	9 (30)	.001
Received treatment, n(%)	2 (5)	19 (17)	13 (43)	<.001
HBsAg loss, n(%)	9 (21)	10 (9)	0 (0)	.010

Data are expressed as median (25th, 75th quartile) or number (percentage) as indicated.

† P<.001,

* P<.05 compared with its baseline level.

‡ P for trend.

A significantly higher frequency of HBsAg loss was observed in the LVL (21%) and FVL (9%) groups, but none in the HVL group. Furthermore, the annualized HBsAg decline was 0.101, 0.067 and 0.040 log_10_ IU/mL in LVL, FVL and HVL groups, respectively (P for trend = .006).

### Genotype C was Associated with More Cirrhosis and HBV-DNA Reduction at End-of-follow-up

HBV genotype B and C patients had comparable baseline age, gender, ALT, HBsAg and HBV-DNA levels [Table 3]. However, genotype C patients had a higher percentage of cirrhosis than genotype B patients either at baseline (16 vs. 5%) or EOF (24 vs. 11%). Genotype C patients had a significantly lower HBV-DNA level (2.74 vs. 3.77 log_10_ IU/mL, P = .041) and HBV-DNA/HBsAg ratio (0.65 vs. 1.40, P = .044) at EOF compared with genotype B. The HBsAg decline during the follow-up was comparable between genotype B and C.

**Table 3:** Characteristics of patients according to HBV genotype B or C.

Characteristics	Genotype B	Genotype C	P value
	n = 126	n = 38	
Age	44 (36, 52)	42 (35, 50)	.317
Male, n(%)	78 (62)	26 (68)	.465
Baseline			
ALT (U/L)	29 (19, 44)	37 (22, 66)	.459
HBsAg (log_10_ IU/mL)	3.35 (2.80, 3.64)	3.62 (3.11, 3.97)	.280
HBV-DNA (log_10_ IU/mL)	3.45 (2.66, 4.56)	3.41 (2.27, 3.90)	.920
HBV-DNA/HBsAg	0.45 (−0.66, 1.54)	0.40 (−1.22, 0.96)	.462
Liver cirrhosis, n(%)	6 (5)	6 (16)	.022
End-of-follow-up			
ALT (U/L)	32 (22, 55)	35 (21, 75)	.132
HBsAg (log_10_ IU/mL)	2.94 (1.83, 3.42)	2.91 (1.64, 3.76)	.727
HBV-DNA (log_10_ IU/mL)	3.77 (2.69, 4.87)	2.74 (1.26, 4.47)	.041
HBV-DNA/HBsAg	1.40 (0.38, 2.51)	0.65 (−0.47, 2.22)	.044
Liver cirrhosis, n (%)	14 (11)	9 (24)	.050
HBsAg decline (log_10_ IU/mL)	0.42 (0.04, 0.99)†	0.55 (0.10, 1.22)†	.173
HBV DNA decline (log_10_ IU/mL)	−0.45 (−1.59, 0.99)	0.47 (−0.09, 1.59)	.065
Duration of FU, year	8.0 (4.7, 11.8)	6.3 (4.3, 10.0)	.105

Data are expressed as median (25th, 75th quartile) or number (percentage) as indicated.

† P<.001 compared with its baseline level.

### Predictors for HBsAg Decline/Loss and Hepatitis Flare

After adjustment for age, gender, genotype, ALT and HBV-DNA level at study entry, baseline HBsAg (1 log_10_ IU/mL increment) independently predicted both HBsAg decline >1 log_10_ IU/mL at EOF (OR: 0.64, 95% CI = 0.43–0.94, P = .023) and HBsAg loss at EOF (OR = 0.37, 95% CI = 0.21–0.65, P = .001) [Table 4]. On the other hand, multivariate analysis showed that male gender (OR = 3.48, 95%CI = 1.14–10.63, P = .028) and baseline HBV-DNA level (1 log_10_ IU/mL increment, OR = 1.50, 95%CI = 1.11–2.03, P = .009) could predict hepatitis flare during the follow-up period. The ROC analysis showed a baseline HBsAg level <50 IU/mL in the LVL group could predict subsequent HBsAg loss with a sensitivity of 82% and specificity of 67%.

**Table 4:** Predictors for HBsAg decline >1 log at end-of-follow-up, HBsAg loss and HBeAg-negative hepatitis by multivariate analysis.

Baseline parameters	HBsAg decline >1 log		HBsAg loss		HBeAg-negative hepatitis	
	OR	95% CI	OR	95% CI	OR	95% CI
Age (1 yr increment)	1.01	0.98–1.05	1.02	0.97–1.08	1.00	0.95–1.04
Male vs. female	1.62	0.73–3.60	1.86	0.44–7.86	3.48*	1.14–10.63
Genotype B vs non-B	0.63	0.27–1.44	0.49	1.12–1.98	1.23	0.41–3.63
ALT (1 U/L increment)	1.00	1.00–1.00	1.00	0.98–1.01	1.00	1.00–1.00
HBsAg (1 log_10_ IU/mL increment)	0.64*	0.43–0.94	0.37†	0.21–0.65	1.50	0.77–2.90
HBV-DNA (1 log_10_ IU/mL increment)	0.86	0.66–1.10	0.99	0.61–1.61	1.50*	1.11–2.03

† P = .001,

* P<.05.

### The Dynamic Change of HBsAg According to Baseline HBsAg Level

**Figure 2:**
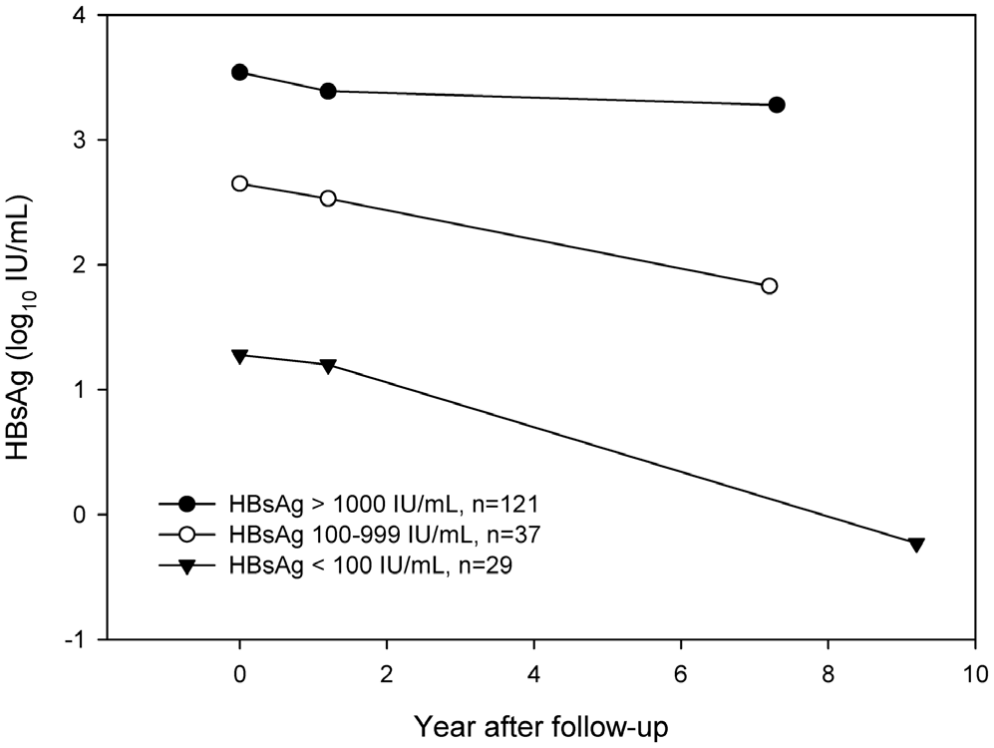
The dynamic change of HBsAg after stratification according to baseline HBsAg level of <100, 100–999 and >1000 IU/mL. The median follow-up duration was 9.2, 7.0 and 7.3 years, respectively. 165 patients (n = 25, 33 and 107 in baseline HBsAg level of <100, 100–999 and >1000 IU/mL) with available first-year HBsAg levels were also plotted. A greater HBsAg decline was observed in patients with lower baseline HBsAg levels (1.22, 0.64, 0.42 log_10_ IU/mL in HBsAg <100, 100–999 and >1000 IU/mL, P for trend = .006).

Patients were further stratified by their baseline HBsAg levels into <100, 100–999 and >1000 IU/mL. The median HBsAg levels were 1.28, 2.65 and 3.54 log_10_ IU/mL at baseline, 1.20, 2.53 and 3.39 log_10_ IU/mL at the first year and −0.23, 1.83 and 3.22 log_10_ IU/mL at EOF [Figure 2]. A significantly greater HBsAg decline was observed in patients with lower baseline HBsAg levels (1.22, 0.64 and 0.42 log_10_ IU/mL, respectively, P for trend = .006). The estimated annualized rate of HBsAg decline was 0.126, 0.091, 0.054 log_10_ IU/mL in those with baseline HBsAg <100, 100–999 and >1000 IU/mL, respectively (P for trend = .014).

## Discussion

In this study, a large cohort of HBeAg-negative treatment-naïve patients was used to demonstrate the longitudinal changes of HBsAg titer. It is known that HBV-DNA level and hepatitis activity fluctuate during the HBeAg-negative phase of chronic HBV infection; therefore, we categorized our patients according to longitudinal HBV-DNA measurements. In agreement with previous results, [8], [14] patients with inactive diseases had significantly lower HBsAg, HBV-DNA level and HBV-DNA/HBsAg ratios, not only at EOF but also at the start of the study recruitment 8 years ago. However, the baseline HBsAg levels were indistinguishable between the FVL and HVL groups, suggesting the predicting role of HBsAg in identifying inactive diseases.

The substantial reduction of HBsAg levels in inactive carriers observed in this study was consistent with the results from genotype D chronic hepatitis B patients, showing that HBsAg was stable in active carriers but declined in inactive ones. [8] The annualized rate of HBsAg decline was reported to be 0.012–0.041 log_10_ IU/mL in active carriers and 0.043–0.077 log_10_ IU/mL in inactive ones. [8], [14] We found this trend similarly in patients with genotype B or C infection. Furthermore, after stratification according to the baseline HBsAg levels, an increasing HBsAg reduction rate was observed in patients with lower baseline HBsAg levels. Taking these lines of evidence together, the annualized rate of HBsAg decline appears non-linear, and may accelerate as the HBsAg level decreases. This information is clinically important for the management of inactive HBV carriers.

It is generally believed that HBsAg level may reflect the “transcriptionally active” cccDNA or the integrated form of HBV genome and is considered to be a surrogate marker of infected hepatocytes. [4] Therefore HBsAg decline indicates a (1) higher clearance and/or (2) a lower production of HBsAg; that is, a reduction of HBsAg mRNA being translated from cccDNA or integrated HBV genome. [21] The dynamics of both mechanisms may contribute to the non-linear and accelerating decline of HBsAg in lower levels. The low HBsAg level suggests host immune taking control over the HBV infection to achieve and sustain an inactive HBV state. [8] In the scenario of acute hepatitis B, the half-life of HBsAg shortened continually after infection: an initial slow decay or non-specific removal of HBsAg, and is followed by the more rapid immunological neutralization with anti-HBs. [22] Besides, a greater decline in HBsAg level is seen after immunomodulatory interferon therapy, compared with direct antiviral nucleos(t)ide analogues. [10], [13], [23] In this study, a lower HBV-DNA/HBsAg ratio was found in LVL and FVL group at baseline and EOF; implicating HBV was less productive in patients with milder liver diseases. [8] Whether the HBsAg decline is associated with inhibition of HBsAg expression and clearance of cccDNA or the integrated form of HBV genome under immunological pressure needs to be clarified by further studies.

HBV genotype is an important viral parameter in predicting disease progression and therapeutic outcome.[24]–[28] In vitro studies demonstrated genotype-specific patterns of intracellular and extracellular HBV-DNA and HBsAg expressions: the HBsAg secretion was most abundant for genotype A followed by B, C and less in D. [29] A genotypic-specific HBsAg decline during pegylated-interferon treatment was also demonstrated, which was higher in genotype A, intermediate in B and D, and lower in C and E. [28], [30] In our study, genotype C patients had a significantly higher percentage of cirrhosis and decline of HBV-DNA, but not HBsAg. This may partly be explained by immunologic attack against HBV-infected hepatocytes, resulting in disease progression to cirrhosis and decline in HBV-DNA but there is still insufficient immune control over HBV infection with HBsAg persistence in genotype C patients. [24], [25] However, further larger studies with special focus on immune functions are needed to validate this important and interesting issue.

Recent studies indicated that a lower HBsAg level can better predict the clinical outcomes, especially HBsAg loss. [6], [31], [32] In this study, we confirmed that HBsAg level positively predicted both HBsAg decline >1 log and HBsAg loss over time, indicating a good immunological control and viral clearance. [14] We found a cut-off HBsAg level <50 IU/mL could predict subsequent HBsAg loss. On the other hand, the predictors for hepatitis flare included male gender and baseline HBV-DNA level, but not HBsAg level. These findings were consistent with our previous data that HBV-DNA levels >/ = 2000 IU/mL can predict HBeAg-negative hepatitis and hepatitis flare among HBeAg seroconverters. [18] Nevertheless, in patients with HBV-DNA <2000 IU/mL (n = 95), HBsAg seemed to predict HBeAg-negative hepatitis flare (OR: 4.29, 95%CI = 0.91–20.27, P = .066) though the statistic power was suboptimal. This fact again supports the complementary role of HBsAg level in low HBV-DNA patients to predict adverse outcomes. In our patients, 10% achieved HBsAg loss during the follow-up and 56% of them developed anti-HBs (>10 mIU/mL) spontaneously. For patients who lost HBsAg without seroconversion to anti-HBs, hepatitis B vaccination might be a strategy to generate anti-HBs. In a recent study, the response rate to the vaccination in prior HBsAg carriers was only 24%. [33] On the other hand, the overall response rate of hepatitis B vaccination in subjects with isolated hepatitis B core antibody was up to 72–86%, [34], [35] and a prior history of HBsAg positivity was associated with a decreased response to vaccination. [36] The reduced response to hepatitis B vaccination was possibly due to the prolonged carriage of HBsAg with ineffective immunity on repeated exposure to HBsAg. Finally, in subjects who have recovered from previous HBV infection, the protection of anti-HBs after HBsAg seroconversion should be cautiously monitored whenever they receive rituximab-containing chemotherapy, because HBsAg seroreversion may occur. [37].

Our study had a few limitations. Due to the retrospective study design, our patients did not have frequent HBV-DNA tests, possibly leading to mis-classification of the disease status. However, the ALT levels were checked regularly, and HBV-DNA would be checked in case of significant abnormal liver function. Furthermore, liver biopsies were not a routine procedure in HBV patients with mild disease. This potential source of error in liver disease status could be partly compensated by serial HBV-DNA testing and regular ultrasonographic examinations.

In summary, patients with inactive HBV infection have a significantly lower HBsAg and HBV-DNA levels during the long-term follow-up than active HBV carriers. The HBsAg decline was significantly greater in inactive carriers with low baseline HBsAg levels, regardless of whether HBV genotype B or C. The HBsAg decline appears non-linear, and accelerates as the HBsAg level lowers. Lower baseline HBsAg level can predict HBsAg decline and loss over time, whereas higher baseline HBV-DNA level can predict HBeAg-negative hepatitis flare.
